# Diagnostic and Prognostic Values of Bile Biomarkers for Malignant Biliary Stenosis

**DOI:** 10.1111/liv.70637

**Published:** 2026-04-21

**Authors:** E. Gigante, V. Untereiner, S. Caruso, R. Rhaiem, L. Grados, C. Boulagnon‐Rombi, S. Adam, G. D. Sockalingum, R. Garnotel, G. Thiefin

**Affiliations:** ^1^ CHU Reims Service D'hépato‐Gastroentérologie et De Cancérologie Digestive Reims France; ^2^ Université De Reims Champagne‐Ardenne BioSpecT‐UR7506 Reims France; ^3^ Université De Reims Champagne‐Ardenne URCATech, PICT Reims France; ^4^ Université Paris Est Créteil INSERM, IMRB Créteil France; ^5^ CHU de Reims Service de Chirurgie Digestive Reims France; ^6^ CHU D'amiens Service De Gastroentérologie Et D'oncologie Digestive Amiens France; ^7^ CHU De Reims Service De Pathologie; Medyc Umr 7369, Université De Reims Champagne‐Ardenne Reims France

**Keywords:** bile biomarkers, cholangiocarcinoma, diagnosis, pancreatic adenocarcioma, prognosis

## Abstract

**Introduction:**

Differentiating between malignant and benign biliary strictures remains a clinical challenge because current diagnostic tools have limited sensitivity. The detection of tumour‐related biomarkers directly in bile, which is in close contact with the lesion, may enhance diagnostic accuracy. This study aimed at evaluating the diagnostic and prognostic values of biliary concentrations of VEGF, PDGF‐AA, IGF and MMPs in patients with extrahepatic cholangiocarcinoma (CCA), pancreatic ductal adenocarcinoma (PDAC), or benign obstruction.

**Methods:**

A total of 100 consecutive patients undergoing ERCP for obstructive jaundice were enrolled and categorised into benign (*n* = 48), CCA (*n* = 23) and PDAC (*n* = 29) groups. Bile samples collected during ERCP were analysed. The diagnostic and prognostic performance of several biomarkers was assessed and, when appropriate, their incremental diagnostic value was assessed in comparison with standard bile cytology.

**Results:**

Biliary concentrations of VEGF, MMP‐1, MMP‐2, MMP‐7 and MMP‐9 were significantly higher in malignant cases than in benign cases. VEGF demonstrated the best diagnostic accuracy (AUROC 0.86, sensitivity 87%, specificity 81%). MMP‐1 and MMP‐7 also showed good diagnostic values (AUROCs 0.79 and 0.81 respectively). In the multivariate analysis, VEGF was independently associated with the diagnosis of malignancy and PDAC, whereas both VEGF and MMP‐2 were independently associated with CCA diagnosis. Using a predefined cut‐off, bile VEGF showed a higher sensitivity than bile cytology, and their combination markedly reduced false‐negative results. Regarding prognosis, higher PDGF‐AA levels were independently associated with better survival rates.

**Conclusion:**

Biliary VEGF is a strong independent biomarker for the diagnosis of malignant biliary obstruction, both in PDAC and CCA. MMP‐2 was independently associated with CCA diagnosis. PDGF‐AA is a promising prognostic biomarker for malignant biliary strictures. These findings support the clinical potential of bile‐based biomarkers for improving the diagnosis and assessing the prognosis of malignant biliary strictures.

## Key Points

- Bile‐based biomarkers, especially VEGF, show strong diagnostic performance in distinguishing malignant from benign biliary obstructions.
- VEGF outperforms standard bile cytology and, when combined with it, significantly reduces false negatives.
- Higher PDGF‐AA levels correlate with improved survival, suggesting a potential prognostic role in malignant biliary disease.

**Abbreviations**
CCA: cholangiocarcinoma
ERCP: endoscopic retrograde cholangiopancreatography
EUS: endoscopic ultrasound
IGF: insulin‐like growth factor
MMP: matrix metalloproteinase
PDAC: pancreatic ductal adenocarcinoma
PDGF: platelet‐derived growth factor
VEGF: vascular endothelial growth factor

## 1 Introduction

The main tumours causing extrahepatic bile duct obstruction are cholangiocarcinoma and adenocarcinoma of the head of the pancreas. Their incidence is increasing in Western countries, and their evolution is rapidly unfavourable when the tumour is not resectable [1, 2, 3]. Differentiating between benign and malignant bile duct strictures may be a difficult and demanding task for clinicians. Guidelines have recently been published by the European Society of Gastrointestinal Endoscopy (ESGE) [4]. Serum tumour markers CA19‐9 and CEA have low accuracy in differentiating malignant from benign obstruction, and their use alone is not recommended, especially in the presence of jaundice. Cytological examination of bile samples obtained via endoscopic retrograde cholangiopancreatography (ERCP) has very low sensitivity and is therefore not recommended [5]. Brush cytology and intraductal biopsies obtained during ERCP have long been the standard for diagnosing malignant common bile duct strictures because of their excellent specificity. However, in a meta‐analysis of nine studies involving 730 patients, the pooled sensitivities of these methods were reported to be only 45% and 48%, respectively, and 59% when combined [6]. Endoscopic ultrasound (EUS)‐guided fine‐needle biopsy, a recently developed diagnostic tool, has been shown to be the most effective method for diagnosing malignant extrahepatic biliary strictures. In a meta‐analysis of 20 studies including 957 patients, the pooled sensitivity and specificity were 80% and 97% respectively [7]. Finally, a combined approach based on tissue sampling by EUS and ERCP has been shown to improve the diagnostic accuracy of each method used. Accordingly, the combination of EUS‐guided fine‐needle biopsy and ERCP‐guided forceps biopsy is currently recommended as the most efficient method for diagnosing extrahepatic biliary strictures [4]. However, its performance is still insufficient to exclude malignancy in the case of negative results [8]. In indeterminate cases, cholangioscopy‐guided biopsies, when available, may improve the diagnostic yield of malignancy. Additional intraductal biliary imaging methods, such as intraductal ultrasound and confocal laser endomicroscopy, are currently in the development stage [4]. Finally, despite complete preoperative evaluation, approximately 7% of patients who underwent surgery for suspected malignant stenosis were postoperatively diagnosed with benign disease [9].

Regarding the quest for novel tumour markers, most studies have been conducted on serum. However, as tumour proliferation occurs in the lumen of the bile ducts in cases of tumour obstruction, analysis of a biliary sample appears to be the most appropriate method for detecting tumour biomarkers linked to the development of these tumours. In addition, bile can be easily collected during biliary drainage, which is a diagnostic and therapeutic procedure for patients with biliary strictures. While several reviews have highlighted the potential of bile‐based biomarkers for the diagnosis of biliary and pancreatic malignancies, the available evidence remains heterogeneous and, to date, has not resulted in validated tools that have modified routine clinical practice [10, 11, 12].

CCA is associated with changes in bile composition, making bile analysis a valuable diagnostic tool. Hydrophobic bile acids, such as deoxycholic acid, may contribute to carcinogenesis, whereas hydrophilic bile acids, such as ursodeoxycholic acid, have protective effects [13].

Contradictory results have been reported regarding the diagnostic value of bile growth factors, such as VEGF and IGF, for the diagnosis of malignant obstruction. Abdel Razik et al. reported that biliary concentrations of IGF‐1 and VEGF were significantly increased in malignant strictures due to CCA or PDAC compared to benign obstruction, and biliary concentrations of these biomarkers were significantly higher in the CCA patients than in pancreatic cancer patients [14]. In contrast, Alvaro et al. did not find any significant difference in VEGF biliary concentrations between patients with extrahepatic cholangiocarcinoma, pancreatic cancer and benign biliary obstruction. However, they reported that IGF‐1 biliary levels may distinguish CCA patients from those with PDAC or benign biliary obstruction [15]. Finally, Navaneethan et al. showed in a pilot study that biliary VEGF‐1 measurement may be a useful diagnostic tool to differentiate pancreatic cancer from CCA and benign biliary stricture [16]. These discrepancies may reflect differences in the studied populations and control group: Abdel‐Razik et al. included North‐African patients, whereas Alvaro and Navaneethan included a substantial proportion of patients with primary sclerosing cholangitis (PSC) among their controls [14, 15, 16]. Regarding PDGF, there are data supporting its role in facilitating the invasion and metastasis of CCA [17, 18, 19, 20], while high expression in tumours from patients with PDAC has been reported to correlate with prolonged survival [21]. The serum concentration of this marker has been reported to be higher in patients with CCA than in those with benign biliary lesions [18, 22], whereas one study in a limited number of patients reported the diagnostic value of a high biliary concentration of PDGF [23].

The significance of matrix metalloproteinases (MMPs) in CCA and PDAC has been primarily examined in surgical specimens and serum. Serum MMP‐7 has been reported to be a valuable diagnostic marker of CCA and PDAC [21, 24, 25]. Data on the diagnostic value of biliary MMPs are limited. In one study, MMP‐9 biliary concentration was significantly higher in patients with CCA than in controls. However, the specificity for the best cut‐off was poor at 32%, making this marker not useful for the diagnosis of CCA in the clinical setting [26]. Finally, other studies have reported that bile cfDNA mutations [27], DNA methylation [28], microRNAs [29] or Pyruvate kinase M2 [30] analysis may identify discriminatory markers to distinguish malignant from benign biliary obstruction. Although promising, the robustness of these results needs to be confirmed by additional prospective studies and external validation before clinical implementation.

Given this, our study aimed at evaluating the diagnostic and prognostic values of biliary VEGF, IGF, PDGF‐AA and MMPs ‐1, ‐2, ‐7 and ‐9 in discriminating between CCA, PDAC and benign obstruction of the common bile duct.

## 2 Patients and Methods

This study included a series of consecutive patients with obstructive jaundice who were referred for endoscopic retrograde cholangiopancreatography (ERCP) at the Hepato‐Gastroenterology Unit of Reims University Hospital between February 2010 and September 2015. Patients with a final diagnosis of benign obstruction, extrahepatic cholangiocarcinoma, or malignant biliary stenosis due to PDAC were included. The exclusion criteria were as follows: age < 18 years, postoperative period of biliary or hepatic surgery, and patients presenting with acute cholangitis. All patients underwent complete clinical, biological and imaging evaluations, including liver tests, ultrasound, abdominal CT, and/or magnetic resonance cholangiopancreatography. The follow‐up period was extended until July 2024.

Endoscopic retrograde cholangiopancreatography (ERCP) was performed to precisely determine the nature of the biliary obstruction and decompress the biliary tract. The examination was performed using a TJF duodenoscope (Olympus Optical, Tokyo, Japan) under general anaesthesia. The bile duct was selectively cannulated using a sphincterotome (Autotome, Boston Scientific, Natick, USA) mounted on a guide wire (Life Partners Europe, Bagnolet, France) without pre‐cut sphincterotomy. Using the guidewire, the cannula was positioned above the stenotic lesion or obstructing biliary stones, and before any injection of contrast material, 5–10 mL of bile was collected by gentle suction with a syringe. Subsequently, the bile ducts were opacified, and the patient was managed according to the endoscopist's decision, following clinical guidelines. Bile samples were sent to the Pathology Laboratory within one to 2 h of collection. Following centrifugation at 1200 × g for 5 min, the cellular pellet was collected, smeared onto slides, fixed and stained for microscopic examination. The remaining supernatant, which was typically discarded as it was no longer needed, was snap‐frozen and stored at −80°C. The final diagnosis of CCA or PDCA was based on ERCP‐guided forceps biopsy and/or EUS‐guided fine needle biopsy, confirmed by the analysis of surgical specimens in patients who underwent surgery.

The following data were retrospectively collected from the medical files: age, sex, serum and bile bilirubin concentrations, and date of death or loss to follow‐up. Survival time was defined as the time between ERCP and death. Patients lost to follow‐up were censored on the date of their last encounter. This retrospective study did not require Ethics Committee approval in accordance with French legislation governing research involving human subjects. Indeed, retrospective studies using information noted in the patients' medical files in daily practice by the medical and nursing staff caring for the patient do not require written informed consent under current French legislation. In compliance with French legislation, information about the study was provided to each patient regarding the type of data being collected and the purposes of the study. Following this information, patients were free to opt out of the study. In the absence of an explicit refusal, participation was considered implied consent. Data were protected and rendered anonymous in compliance with the French and European legislation.

## 3 Measurement of Bile Biomarkers

After thawing the bile supernatant, the following human biomarkers were measured using DuoSet ELISA Development systems (R&D Systems, Lille, France): MMP‐1 (DY901), ‐2 (DY902), ‐7 (DY907), ‐9 (DY909), VEGF (DY293B), IGF (DY291) and PDGF‐AA (DY221). Assays for each biomarker were performed according to the manufacturer's instructions. The measurements were performed with an adapted dilution of the samples (2‐ to 500‐fold dilution) realised in 0.9% NaCl (Miniversol, Dutscher, France), except for MMP‐2 and VEGF, which were measured without dilution. Additional technical procedures are described in the Supporting Information.

## 4 Statistical Analysis

Statistical analysis and graphical representation of data were performed using statistical software R (version 4.0.2) (R Foundation for Statistical Computing, Vienna, Austria), Bioconductor packages (version 3.4), statistical software MedCalc (version 22.007) and GraphPad Prism (version 5.00). Quantitative variables were expressed as medians and interquartile ranges (IQR) and were compared using nonparametric tests (Mann–Whitney test or Kruskal–Wallis test with Dunn's multiple comparison test when appropriate). Qualitative data were analysed using the chi‐square test. The diagnostic effectiveness of the various biomarkers was assessed using the area under the receiver operating characteristic curves (AUC) and the corresponding 95% confidence intervals (CI). Cut‐off values were determined based on those that maximized the Youden index. For each specified cut‐off value, we documented the sensitivity and specificity, accompanied by 95% CIs. Univariate and multivariate logistic regression were performed to identify independent diagnostic biomarkers. Overall survival was defined as the duration from ERCP to death. To appraise the prognostic performance, the expression of biomarkers was discretised by selecting a cut‐off that exhibited the highest discriminative power in relation to survival. The optimal cut‐off point was derived using the maximally selected rank statistics, as implemented in the maxstat R package. Survival curves were plotted using the Kaplan–Meier method and compared using log‐rank statistics. Univariate and multivariate analyses were conducted using the Cox proportional hazards regression model.

### 4.1 Declaration of Generative AI and AI‐Assisted Technologies in the Writing Process

During the preparation of this work, the authors used PaperPal, Deepl and Chat‐GPT in order to rephrase and correct the text. After using this tool/service, the authors reviewed and edited the content as needed and took full responsibility for the content of the publication.

## 5 Results

### 5.1 Characteristics of Patient Population

One hundred patients were included and divided into a benign group (*n* = 48), CCA group (*n* = 23) and PDAC group (*n* = 29). The benign group comprised 46 patients with obstructive stones in the common bile duct and two patients with benign biliary stricture (one due to chronic pancreatitis and the other due to previous surgical bile wound). Patients in the CCA group had distal CCA (from the common bile duct, *n* = 15) or perihilar CCA (from the hepatic ducts, *n* = 8). Those in the PDAC group had malignant stenosis of the common bile duct related to the local development of head pancreatic carcinoma. As shown in Table 1, there was a predominance of male patients in the CCA and PDAC groups (78.3% and 58.6% respectively), whereas the sex ratio was equilibrated in the group of patients with benign obstruction (47.9%). Patients with benign obstruction were slightly older than those in the CCA and PDAC groups (median age 73 years vs. 66 and 69 years respectively). All patients were jaundiced at enrolment, but jaundice was more severe in the malignant groups than in the benign group (median serum bilirubin concentrations of 271 and 207 μmol/L in the PDAC and CCA groups versus 65 in the benign group, *p* < 0.001). In contrast, the bilirubin levels in bile were lower in malignant groups than in the benign group, reaching the significance level for CCA compared to the benign group (median concentrations of 23 μmol/L in the CCA group versus 656 μmol/L in the benign group, *p* < 0.05). Serum concentrations of cholestasis markers gamma‐glutamyltransferase and alkaline phosphatase were significantly increased in malignant groups, PDAC and CCA, compared to those in the benign group (Table 1).

**TABLE 1:** Patient characteristics in benign and malignant groups.

	Benign group	PDAC group	CCA group	*p* ^1^	PDAC + CCA group	*p* ^2^
*n*	48	29	23		52	
Sex, male, *n* (%)	23 (47.9)	17 (58.6)	18 (78.3)	0.05	35 (67.3)	0.068
Age, years (Median [IQR])	73 [62.25, 80.50]	69 [59, 73]	66 [59, 73.5]	0.1	66.5 [58.75, 73.25]	0.036
Serum biliribin, μmol/L (Median [IQR]) Missing data	65 [16, 120] 0	271*** [185, 340] 0	207*** [175, 391] 2	< 0.001	260 [178, 356] 2	< 0.001
Bile biliribin, μmol/L (Median [IQR])	656 [303, 1194]	235^ns^ [11, 1112]	23* [4, 506]	0.011	69 [7.7, 718]	0.008
Serum AST, IU/L (Median [IQR]) Missing data	74 [40, 169] 2	131^ns^ [86, 190] 1	107^ns^ [80, 203] 2	0.075	122 [82, 188] 3	0.011
Serum ALT, IU/L (Median [IQR]) Missing data	102 [52, 332] 2	219^ns^ [83, 285] 1	94^ns^ [69181] 2	0.36	136 [73, 265] 3	0.55
Serum γGT, IU/L (Median [IQR]) Missing data	311 [119, 719] 2	759*** [442, 1248] 0	581* [262, 1253] 2	0.0004	709 [3691242] 2	< 0.0001
Serum ALP, IU/L (Median [IQR]) Missing data	222 [96, 302] 0	581*** [428, 740] 0	381** [331, 609] 2	< 0.0001	495 [369, 683] 2	< 0.0001
Median follow‐up, months	41	7***	15**	< 0.0001	10.5	< 0.0001
Median survival, months	79	7***	15***	< 0.0001	10.5	< 0.0001

*Note:* Comparisons between PDAC and CCA groups were not significant for all variables except for median follow‐up and median survival (*p* = 0.036, log‐rank‐test). Significant levels for pairwise comparisons (post hoc Dunn's multiple comparison test, χ² test, log‐rank test) were the following.

^1^ *p*‐value for comparison between benign, PDAC and CCA groups (Kruskal‐Wallis test, χ² test, log‐rank test).

^2^ *p*‐value for comparison between benign and PDCA+CCA groups (Mann‐Whitney test, χ² test, log‐rank test).

* *p* < 0.05 versus benign group.

** *p* < 0.01 versus benign group.

*** *p* < 0.001 versus benign group.

^ns^ Not significant versus benign group.

### 5.2 Comparison of Biliary Concentrations of MMPs, VEGF, PDGF and IGF in Benign and Malignant Groups

The biliary concentrations of MMP‐1, MMP‐2, MMP‐7, MMP‐9 and VEGF were significantly higher in the malignant group, including both PDAC and CCA, than in the benign group (Table 2). Multiple pairwise comparisons between groups showed that MMPs and VEGF were significantly higher in the CCA group and PDAC group than in the benign group, except for MMP‐9 and MMP‐2 for the PDAC group (Table 2 and Figure S1). Biliary concentrations of PDGF‐AA were also higher in the malignant group, including CCA and PDAC, than in the benign group (0.56 ng/mL vs. 0.37 ng/mL, *p* = 0.026). However, the difference did not reach significance when the malignant groups were analysed separately. No significant differences were observed in the concentrations of any of the biomarkers between the PDAC and CCA groups (Table 2 and Figure S1). This trend was confirmed by PCA analysis. The lack of difference was also confirmed by the multivariable analysis comparing PDAC and CC (Figure S2 and Table S1).

**TABLE 2:** Comparison of MMPs, VEGF, PDGF‐AA and IGF biliary concentrations in benign and malignant groups. All concentrations are expressed as nanograms per millilitre.

	Benign group	PDAC group	CCA group	*p* ^1^	PDAC + CCA group	*p* ^2^
*N*	48	29	23		52	
MMP‐1 (Median [IQR])	2.19 [0.21–16.26]	30.76*** [16.42–43.28]	18.80** [11.81–37.34]	< 0.001	25.47 [13.68–41.25]	< 0.001
MMP‐2 (Median [IQR]) Missing data	0.56 [0.30–1.60] 0	1.67^ns^ [0.38–2.40] 0	1.72* [0.66–3.78] 1	0.012	1.67 [0.52–2.83] 1	0.005
MMP‐7 (Median [IQR])	28.25 [6.10–82.62]	142.70*** [95.01–360.87]	121.30** [79.31–316.70]	< 0.0001	129.45 [82.37–344.14]	< 0.001
MMP‐9 (Median [IQR])	1.65 [0.12–6.91]	3.55^ns^ [1.49–6.85]	6.90* [2.12–62.00]	0.023	4.55 [1.61–18.72]	0.024
VEGF (Median [IQR])	0.13 [0.00–0.48]	1.40*** [0.79–1.66]	1.74*** [0.64–3.22]	< 0.001	1.521 [0.78–2.02]	< 0.001
PDGF‐AA (Median [IQR]) Missing data	0.37 [0.06–0.60] 9	0.57^ns^ [0.23–1.07] 3	0.52^ns^ [0.33–0.99] 7	0.083	0.56 [0.26–1.05] 10	0.026
IGF (Median [IQR]) Missing data	0.61 [0.02–1.12] 9	0.25^ns^ [0.04–0.75] 3	0.05^ns^ [0.00–0.61] 7	0.17	0.13 [0.02–0.73] 10	0.217

*Note:* Significant levels of the post hoc Dunn's multiple comparison test were the following.

^1^ *p*‐value for comparison between benign, PDAC and CCA groups (Kruskal–Wallis test).

^2^ *p*‐value for comparison between benign and PDCA + CCA groups (Mann–Whitney test). Comparisons between PDAC and CCA groups were not significant for all biomarkers.

* *p* < 0.05 versus benign group.

** *p* < 0.01 versus benign group.

*** *p* < 0.001 versus benign group.

^ns^ not significant versus benign group.

### 5.3 Diagnostic Performance of Biliary Biomarkers

**FIGURE 1:**
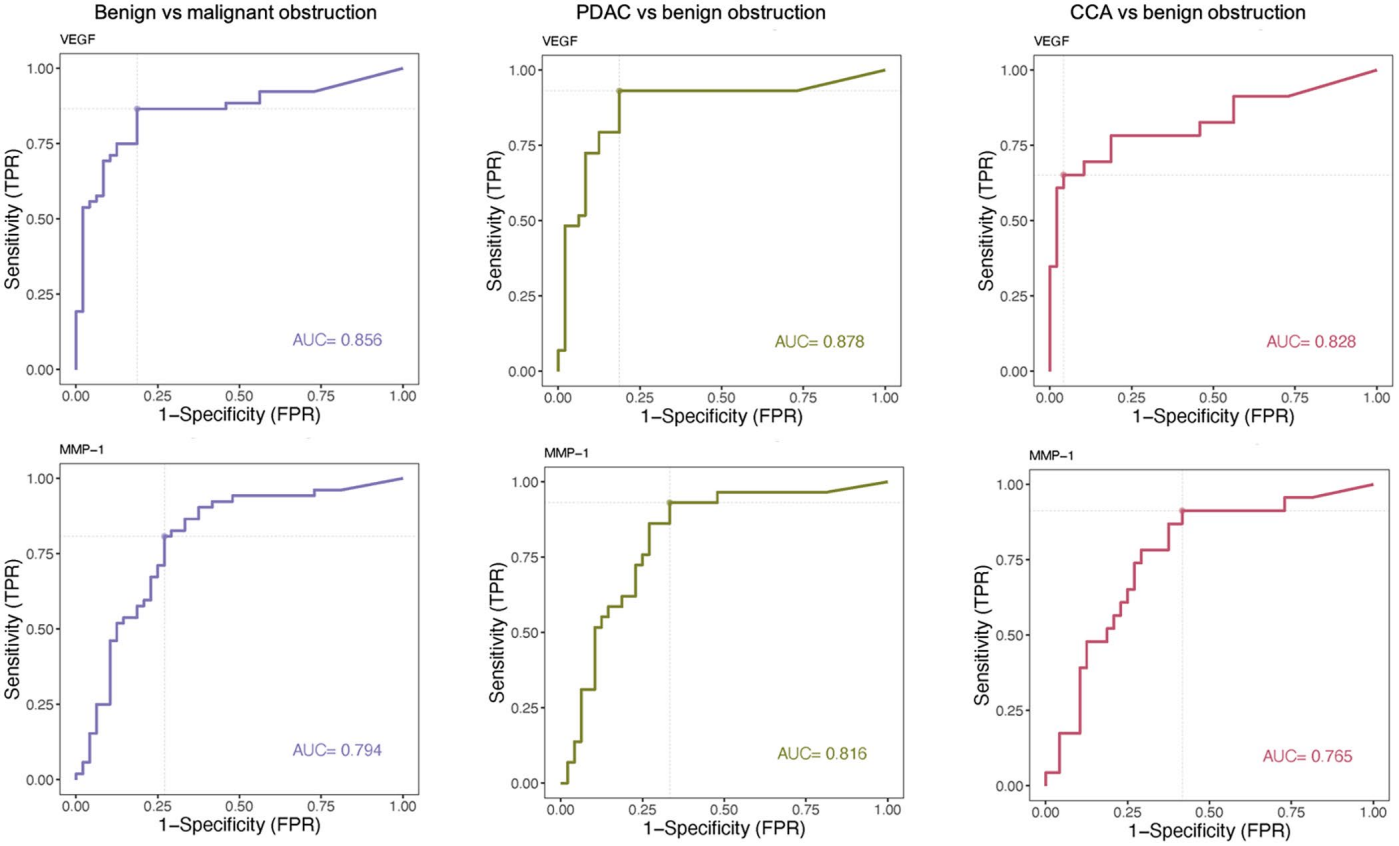
ROC curves of VEGF and MMP‐1 values in differentiating patients with malignant versus benign obstruction, pancreatic ductal adenocarcinoma (PDAC) versus benign obstruction and cholangiocarcinoma (CCA) versus benign obstruction.

The diagnostic performance of biliary markers in differentiating malignant from benign obstructions was analysed using the ROC curve method (Table 3 and Figure 1).

**TABLE 3:** Diagnostic performance of different biliary biomarkers in identifying specific clinical conditions, such as benign versus malignant biliary obstruction, pancreatic ductal adenocarcinoma (PDAC) versus benign obstruction, and cholangiocarcinoma (CCA) versus benign obstruction. Each biomarker was evaluated according to the area under the ROC curve (AUROC), optimal cut‐off, sensitivity and specificity with associated confidence intervals. All cut‐off concentrations are expressed in nanograms per millilitre (ng/mL). AUROCs for IGF were not calculated in PDCA and CCA subgroups due to insufficient numbers of patients.

Variable	AUROC	CI	Best cut‐off	Sensitivity	CI	Specificity	CI
Comparison of malignant versus benign obstruction							
MMP‐1	0.79	0.71–0.88	10.49	0.81	0.67–0.90	0.73	0.58–0.85
MMP‐7	0.81	0.73–0.90	76.62	0.81	0.67–0.90	0.73	0.58–0.85
VEGF	0.86	0.78–0.93	0.60	0.87	0.74–0.94	0.81	0.67–0.91
MMP‐2	0.66	0.56–0.77	0.67	0.71	0.56–0.83	0.58	0.43–0.72
MMP‐9	0.63	0.52–0.74	0.45	0.87	0.74–0.94	0.48	0.33–0.63
PDGF	0.64	0.52–0.76	0.23	0.81	0.66–0.91	0.44	0.28–0.60
IGF	0.42	0.30–0.55	—	—	—	—	—
Comparison PDAC versus benign obstruction							
MMP‐1	0.82	0.71–0.92	5.52	0.93	0.77–0.99	0.67	0.52–0.80
MMP‐7	0.82	0.72–0.93	76.77	0.83	0.64–0.94	0.73	0.58–0.85
VEGF	0.88	0.79–0.97	0.60	0.93	0.77–0.99	0.81	0.67–0.91
MMP‐2	0.63	0.50–0.76	0.67	0.69	0.49–0.85	0.58	0.43–0.72
MMP‐9	0.58	0.45–0.71	0.45	0.83	0.64–0.94	0.48	0.33–0.63
PDGF	0.64	0.50–0.78	0.55	0.58	0.37–0.77	0.69	0.52–0.83
Comparison CCA versus benign obstruction							
MMP‐1	0.76	0.64–0.89	3.75	0.91	0.72–0.99	0.58	0.43–0.72
MMP‐7	0.80	0.69–0.92	76.62	0.78	0.56–0.93	0.73	0.58–0.85
VEGF	0.83	0.72–0.94	1.43	0.65	0.43–0.84	0.96	0.86–0.99
MMP‐2	0.71	0.57–0.85	0.60	0.82	0.60–0.95	0.52	0.37–0.67
MMP‐9	0.69	0.56–0.83	0.22	1.00	0.85–1.00	0.42	0.28–0.57
PDGF	0.65	0.49–0.82	0.25	0.88	0.62–0.98	0.44	0.28–0.60

VEGF emerged as the best diagnostic biomarker for distinguishing malignant from benign obstruction, with an area under the ROC curve (AUROC) of 0.86. The best cut‐off had a sensitivity of 87% and specificity of 81%. MMP‐7 and MMP‐1 had AUROCs of 0.81 and 0.79, respectively, both with a cut‐off sensitivity of 81% and specificity of 73%. In contrast, MMP‐2, MMP‐9, PDGF‐AA and IGF had poor diagnostic interest with an AUROC below 0.70 (Table 3 and Figure S3).

For PDAC diagnosis, three biliary biomarkers (MMP‐1, MMP‐7 and VEGF) had an AUROC greater than 0.80, which is considered clinically useful. The best diagnostic biomarker was VEGF, with a cut‐off of 0.60 ng/mL, which predicted PDAC with 93% sensitivity and 81% specificity. MMP‐1 and MMP‐7 both showed an AUROC of 0.82. However, the best cut‐off for MMP‐1 had a higher sensitivity (93%) than that of MMP‐7 (83%), whereas the specificity of the MMP‐7 cut‐off was slightly better (73% vs. 67% for MMP‐1). The markers MMP‐2, MMP‐9 and PDGF‐AA performed less well, with AUROCs of 0.63, 0.58 and 0.64 respectively.

For the diagnosis of CCA, VEGF was also the best biomarker, with an AUROC of 0.83 and a cut‐off of 1.43 ng/mL, allowing it to predict CCA with a specificity of 96%. However, the sensitivity was relatively low (65%), indicating a higher likelihood of false negatives. MMP‐7 and MMP‐1 also showed AUROC of 0.80 and 0.76 respectively. The best cut‐off for MMP‐1 had a sensitivity of 91% but a lower specificity (58%), whereas the best cut‐off for MMP‐7 had a sensitivity of 78% and a specificity of 73%.

Using a VEGF cut‐off of > 0.6 ng/mL, the sensitivity for malignancy detection was 87% in the whole cohort (PDAC+CCA) versus 54% for bile cytology (*p* = 0.0003), with a similarly marked improvement in PDAC (93% vs. 48%, *p* = 0.0003) and a nonsignificant increase in CCA (78.3% vs. 60.9%, *p* = 0.20) (Table 4).

**TABLE 4:** Comparison of sensitivity of bile VEGF measurement and bile cytologic analysis in the whole population and different cancer subgroups.

	VEGF > 0.6 ng/mL	Bile cytology	*p*
Whole population (PDAC + CCA), *n* = 52			
True positive	45	28	
False negative	7	24	
Sensitivity	87%	54%	0.0003*
PDAC patients (*n* = 29)			
True positive	27	14	
False negative	2	15	
Sensitivity	93%	48%	0.0003**
CCA patients (*n* = 23)			
True positive	18	14	
False negative	5	9	
^2^Sensitivity	78.3%	60.9%	**0.20** *

* χ² test.

** Fisher's exact test.

Bile VEGF and cytology showed complementary diagnostic profiles. When combined, sensitivity reached 96.2% in the overall cohort, 100% in PDAC and 91.3% in CCA, highlighting the added value of bile VEGF in reducing false‐negative results of standard cytology (Table S1).

### 5.4 Correlations Between Biomarkers and Serum and Bile Bilirubin Levels

Serum bilirubin concentration appeared to have a moderate positive relationship with MMP‐7 (0.39), indicating that, as cholestasis worsens, the levels of biliary MMP‐7 increase. A poor positive correlation was observed between serum bilirubin and MMP1 (0.23) and VEGF (0.25). In addition, serum bilirubin showed only weak positive correlations with PDGF (0.10) and MMP‐2 (0.08) and a negligible correlation with MMP‐9 (−0.01). Negative correlations were observed with IGF (−0.15), age (−0.23) and bile bilirubin concentration (−0.26). The only notable links for biliary bilirubin concentration were a weak negative correlation with serum bilirubin concentration (−0.26) and a weak positive correlation with IGF (0.16) (c.f. Figure S4).

### 5.5 Univariate and multivariate logistic regression analysis of biliary biomarkers associated with malignant tumour, CCA or PDAC compared to benign obstruction

Univariate and multivariate logistic regression analyses identified the main factors associated with malignant tumours, CCA or PDAC (Table 5). Among the variables analysed, serum bilirubin and VEGF emerged as significant and independent predictors. In the multivariate analysis, serum bilirubin and VEGF maintained their significance with an OR of 1.01 (95% CI: 1.00–1.02, *p* = 0.0026) and 5.0510 (95% CI: 1.2157–20.9850, *p* = 0.0258), respectively, in the comparison between the malignant versus benign groups. The other variables taken into account in the analysis, including age, sex, biliary bilirubin, MMP‐9, IGF, MMP‐1, MMP‐2 and PDGF‐AA, showed no relevant associations or lost significance in the multivariate analysis (Table 5).

**TABLE 5:** Univariate and multivariate analyses: Variable association with malignant tumour, CCA or PDAC compared to benign obstruction.

Variable	Available data	Univariate *p*	Univariate OR	Univariate CI	Multivariate *p*	Multivariate OR	Multivariate CI
Malignant versus benign obstruction							
Male sex	100	0.0513	2.2379	0.9953–5.0315	—	—	—
Age	100	0.4865	0.9903	0.9636–1.0178	—	—	—
Serum bilirubin	96	< 0.0001	1.0130	1.0077–1.0183	0.0026	1.0113	1.0039–1.0188
Bile bilirubin	100	0.3955	0.9998	0.9994–1.0002	—	—	—
MMP‐1	100	0.0005	1.0465	1.0201–1.0735	0.1733	0.9709	0.9305–1.0131
MMP‐7	100	0.0001	1.0117	1.0058–1.0175	0.6664	1.0016	0.9945–1.0087
VEGF	100	< 0.0001	8.4519	3.5974–19.8572	0.0258	5.0510	1.2157–20.9850
MMP‐2	99	0.0099	1.3918	1.0826–1.7892	0.3439	1.2173	0.8101–1.8292
MMP‐9	100	0.5450	1.0009	0.9980–1.0038	—	—	—
PDGF‐AA	81	0.0244	3.0598	1.1557–8.1006	0.2773	2.0060	0.5713–7.0439
IGF	81	0.5270	0.9288	0.7387–1.1677	—	—	—
PDAC versus benign obstruction							
Male sex	77	0.3635	1.5399	0.6069–3.9068	—	—	—
Age	77	0.6453	0.9931	0.9643–1.0228	—	—	—
Serum bilirubin	77	< 0.0001	1.0147	1.0082–1.0213	0.0536	1.0091	0.9999–1.0185
Bile bilirubin	77	0.9248	1.0000	0.9995–1.0005	—	—	—
MMP‐1	77	0.0011	1.0452	1.0177–1.0734	0.1429	0.9634	0.9165–1.0127
MMP‐7	77	0.0003	1.0117	1.0053–1.0182	0.4252	1.0030	0.9957–1.0103
VEGF	77	< 0.0001	13.8233	4.5877–41.6512	0.0466	6.3658	1.0286–39.3962
MMP‐2	77	0.0471	1.3251	1.0036–1.7496	0.7389	1.0731	0.7086–1.6252
MMP‐9	65	0.5826	0.9985	0.9932–1.0038			
PDGF‐AA	65	0.0344	3.0371	1.0852–8.4995	0.2452	2.2544	0.5724–8.8795
IGF	77	0.9389	1.0000	0.9998–1.0002	—	—	—
CCA versus benign obstruction							
Male sex	71	0.0191	3.913	1.2499–12.2504	0.4057	4.2590	0.1400–129.6052
Age	71	0.5400	0.9908	0.9613–1.0212	—	—	—
Serum bilirubin	67	0.0003	1.099	1.0046–1.0152	0.0083	1.0107	1.0028–1.0188
Bile bilirubin	71	0.0460	0.999	0,9980–1.0000	0.8100	0.9998	0.9983–1.0013
MMP‐1	71	0.0230	1.0312	1.0042–1.0588	0.4784	0.9894	0.9607–1.0190
MMP‐7	71	0.0006	1.0105	1.0045–1.0165	0.8304	1.0013	0.9896–1.0131
VEGF	71	0.0002	4.9804	2.1219–11.6896	0.0309	4.0398	1.1368–14.3566
MMP‐2	70	0.0089	1.4880	1.1050–2.0038	0.0451	2.1991	1.0174–4.7537
MMP‐9	71	0.2259	1.0021	0.9987–1.0054	—	—	—
PDGF‐AA	55	0.0794	3.2154	0.8720–11.8557	—	—	—
IGF	55	0.1115	0.3490	0.0955–1.2762	—	—	—

In multivariable analysis, VEGF remained the only independent variable associated with PDAC, showing an OR of 6.36, with a 95% CI ranging from 1.0286 to 39.3962, and a *p* value of 0.0466. In contrast, total serum bilirubin, although significant in the univariate analysis (*p* < 0.0001), did not maintain statistical significance in the multivariate analysis (*p* = 0.053) (Table 5).

Few variables were independently associated with the diagnosis of CCA: serum bilirubin (*p* = 0.008, OR = 1.01), VEGF (*p* = 0.031, OR = 4.0398) and MMP‐2 (*p* = 0.045, OR = 2.19). In contrast, factors such as biliary bilirubin, MMP‐1, MMP‐7 and male sex lost significance in the multivariate model (Table 5).

### 5.6 Prognostic Values of Biliary Biomarkers in Patients With CCA or PDAC

In the univariate analysis, the CCA group showed a hazard ratio (HR) of 0.5627 (95% CI: 0.3200–0.9895) with a *p* value of 0.0459 (Table 6). In the multivariate analysis, the HR was 0.5992 (95% CI: 0.3113–1.1536), but the *p >* 0.1254, indicating that the association was no longer statistically significant (Table 6).

**TABLE 6:** Univariate and multivariate analysis for overall survival.

	Available data	Univariate			Multivariate		
		HR	CI	*p*	HR	CI	*p*
CCA (baseline: PDAC)	52	0.5627	0.3200–0.9895	0.0459	0.5992	0.3113–11 536	0.1254
Male sex (baseline: Female)	52	1.1048	0.6088–20 048	0.7431	—	—	—
Age	52	1.0234	0.9918–1.0559	0.1480	—	—	—
Serum bilirubin	50	1.0001	0.9982–1.0021	0.8870	—	—	—
Bile biliribin	52	1.0001	0.9998–1.0003	0.5606	—	—	—
MMP‐1	52	1.0056	0.9982–1.0130	0.1416	—	—	—
MMP‐7	52	1.0005	0.9987–1.0023	0.5963	—	—	—
VEGF	52	0.9769	0.8054–1.1851	0.8129	—	—	—
MMP‐2	51	0.9979	0.8900–1.1190	0.9718	—	—	—
MMP‐9	52	0.9996	0.9979–1.0012	0.6078	—	—	—
PDGF‐AA	42	0.5843	0.3624–0.9420	0.0274	0.5189	0.3026–0.8900	0.0172
IGF	42	1.1006	0.9394–1.2894	0.2354	—	—	—

For PDGF‐AA, the univariate analysis indicated a hazard ratio of 0.5843 (CI: 0.3624–0.9420) with a *p* value of 0.0274, which was statistically significant (Table 6). In the multivariate analysis, the hazard ratio was nearly the same at 0.5189 (CI: 0.3026–0.8900), and the *p <* 0.0172, still showing statistical significance (Table 6).

**FIGURE 2:**
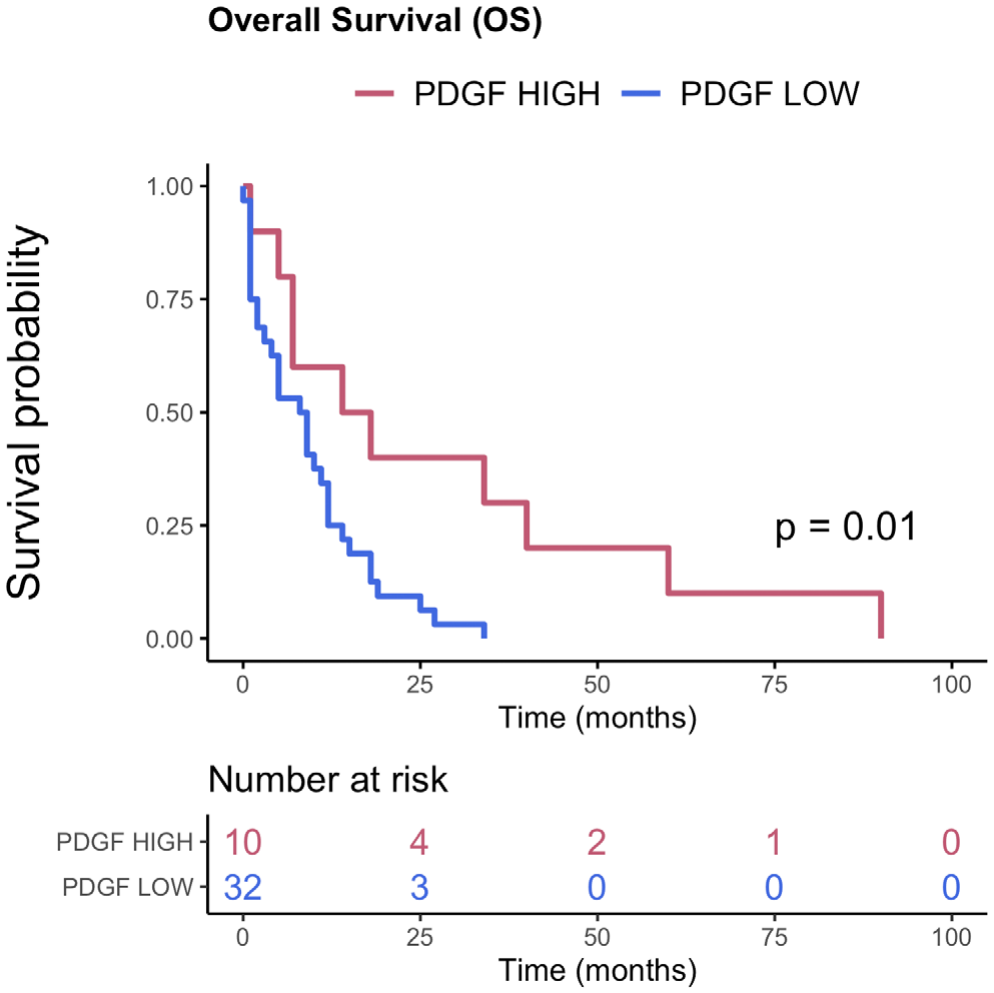
Overall survival according to biliary PDGF levels in malignant group: PDGF HIGH > 1.078 ng/mL and PDGF LOW < 1.078 ng/mL.

The other parameters analysed, such as MMP‐1, MMP‐7, VEGF, MMP‐2, MMP‐9, serum bilirubin and biliary bilirubin, did not show significant associations in the univariate analysis, with HR close to 1 and high *p* values, indicating that they were not significantly correlated with overall survival (Figure 1 and Table 6). After determining the optimal cut‐off value for PDGF‐AA levels (1.078 ng/mL), patients were stratified into two groups: PDGF HIGH (elevated PDGF levels) and PDGF LOW (low PDGF levels). Figure 2 illustrates the overall survival curves for the two groups, demonstrating a significant difference in the survival outcomes. Specifically, patients with high PDGF levels (PDGF HIGH, red line) had a significantly higher probability of survival than those with low PDGF levels (PDGF LOW, blue line). This difference is supported by a *p* value of 0.01, which confirms the statistical significance.

As shown in the graph, the survival of PDGF LOW patients declined rapidly in the first 25 months, with all patients dying within this period. In contrast, in the PDGF HIGH group, survival was longer, with some patients surviving for more than 75 months.

## 6 Discussion

This study was based on the working hypothesis that biomarkers of malignant proliferation inside the biliary tract would be more efficiently identified in bile than in serum, as bile is in direct contact with malignant cells. We selected some molecules: MMPs, VEGF, PDGF‐AA and IGF‐1, whose interest has previously been suggested as serum biomarkers for the diagnosis of various carcinomas, including CCA and PDAC. Analysis of biliary biomarkers revealed significant associations with malignant tumours. In our study, biliary VEGF emerged as an independent predictor of malignancy in both univariate and multivariate analyses. While some biomarkers, such as MMP‐1, ‐7, ‐9 and PDGF, showed initial promise in univariate analysis, they lost significance in multivariate models. Interestingly, biliary MMP‐2 was significantly associated with an increased risk of CCA but not PDAC.

In addition, bile VEGF measurement demonstrated a significantly higher sensitivity than standard bile cytology for the detection of pancreatobiliary malignancies. Using a cut‐off value of > 0.6 ng/mL, bile VEGF achieved a sensitivity of 87% in the overall cohort (PDAC + CCA), clearly outperforming cytology (54%). This diagnostic advantage was particularly evident in PDAC patients, where VEGF sensitivity reached 93% compared to 48% for cytology, while in CCA patients, the increase (78.3% vs. 60.9%) did not reach statistical significance. Importantly, bile VEGF and cytology showed complementary diagnostic profiles. When the two methods were combined, sensitivity increased to 96.2% in the whole cohort, reached 100% in PDAC and 91.3% in CCA, substantially reducing the false‐negative rate associated with cytology alone. These findings suggest that bile VEGF may represent a valuable adjunctive biomarker to standard cytologic analysis, improving diagnostic yield in suspected pancreatobiliary malignancies, particularly in cases with negative or inconclusive cytology. The only molecule in bile with prognostic value in the multivariable analysis for survival in patients with malignant biliary stenosis was PDGF.

VEGF plays a crucial role in neoangiogenesis and is actively involved in the growth and metastatic spread of different types of cancer, including CCA and PDAC. Immunohistochemical studies have shown that VEGF is a prognostic marker in resected primary tumour specimens from patients with extrahepatic CCA and PDAC [31, 32]. Contradictory results have been reported regarding the diagnostic and prognostic values of serum VEGF in CCA and PDAC [14, 15, 21, 33]. Our study focused on the value of biliary biomarkers. We found that biliary VEGF concentration was an independent diagnostic factor for malignant obstruction due to CCA or PDAC, with an AUROC of 0.86. At the cut‐off of 0.6 ng/mL, the sensitivity was 87%, and the specificity was 81%, indicating excellent diagnostic performance to distinguish malignant from benign obstruction. However, biliary VEGF levels did not allow differentiation between patients with PDAC and CCA. These results are in agreement with the study by Abdel Razic et al. [14]. In a series of 109 patients, including 47 with malignant biliary obstruction due to PDAC or CCA and 62 with benign obstruction, these authors showed that biliary VEGF levels were significantly higher in malignant than in benign cases. Using a cut‐off value of 0.5 ng/mL, the sensitivity and specificity were 90.3% and 84.9%, respectively, with an AUROC of 0.91, which is very close to our results. Similarly, in a pilot study including 15 patients with PDAC, 29 with benign obstruction, and only nine with CCA, Navaneethan et al. reported the diagnostic value of biliary VEGF for the diagnosis of malignant biliary obstruction due to PDAC compared to benign biliary conditions. At a cut‐off of 0.5 ng/mL, the sensitivity and specificity of biliary VEGF were 93.3% and 72.7%, respectively, with an AUROC of 0.89. In this study, biliary concentrations of VEGF did not allow discrimination between CCA patients and patients with benign obstruction. However, this result should be interpreted with caution, given the small number of CCA patients [16]. In contrast, in a series of 73 patients with biliary obstruction due to PDAC (*n* = 29), CCA (*n* = 29) and benign abnormalities (*n* = 25), Alvaro et al. did not observe a significant difference in biliary VEGF levels between the three groups [15].

Our study reported that biliary VEGF concentration was very weakly positively correlated with serum bilirubin level (Rho = 0.25) and not correlated with bile bilirubin level (Rho = −0.1), indicating that biliary VEGF level is mostly independent of the degree of cholestasis. Contradictory results have been reported in the literature, with one study reporting a good correlation between biliary VEGF and serum bilirubin levels [14] and another study showing no correlation [16]. None of these studies considered the level of bilirubin in bile in the correlation analysis.

Matrix metalloproteinases are a family of proteolytic enzymes playing an important role in tumour angiogenesis, tumour invasion and metastasis through degradation of the extracellular matrix. Their diagnostic and prognostic values have been investigated in surgical specimens and the serum of patients with CCA or PDAC. Several MMPs expressed in tumour tissues have been shown to predict a poor postoperative survival: MMP‐2, ‐7 and ‐9 in hilar or extrahepatic bile duct CCA [19, 34] and MMP‐2, ‐7, ‐9 and ‐11 in resectable PDAC, although conflicting results have been reported [35, 36, 37].

Regarding the value of serum concentrations, MMP‐7 and MMP‐9 have been reported as valuable diagnostic markers of CCA and PDAC. In a series of 59 patients with CCA, mostly perihilar and 128 patients with obstructive jaundice related to benign biliary diseases, MMP‐7 levels were significantly higher in CCA patients than in controls and outperformed CA19‐9 in diagnostic accuracy (AUC 0.84 vs. 0.79) [38]. Similar results were reported by Lumachi et al. [25]. Kahlert et al. identified that MMP‐1, ‐3, ‐7, ‐9, ‐10 and ‐12 were upregulated in the serum of patients with PDAC, with MMP‐7 and ‐12 being the most effective diagnostic biomarkers.

Data on the diagnostic value of biliary MMPs are limited. In a series of 33 patients with CCA and 80 patients with choledocholithiasis, Ince et al. showed significantly higher biliary levels of MMP‐9 in CCA cases [26]. In this study, the diagnostic reliability of biliary MMP‐9 was moderate, with an AUROC of 0.74 [26]. Our study is the first to provide information on the diagnostic value of a panel of biliary MMPs. We showed that the biliary concentrations of MMP‐1, ‐2, ‐7 and ‐9 were significantly higher in the PDAC and CCA groups than in the benign group. Matrix metalloproteinase‐1 and ‐7 had the best diagnostic performance with AUROC of 0.82 (both MMPs) for PDAC and 0.76 and 0.80, respectively, for CCA, whereas MMP‐2 and ‐9 had moderate to poor diagnostic performance, with AUC values of 0.63 and 0.58, respectively, for PDAC and 0.71 and 0.69, respectively, for CCA. In the multivariate analysis, the only independent predictive marker among MMPs was MMP‐2 for the diagnosis of CCA, with an OR of 2.20 (1.02–4.75). However, this marker was not effective in diagnosing PDAC. Our results are in accordance with those of a study reporting the association of tumoral expression of MMP‐2 in extrahepatic CCA with higher expression in cases of neural invasion [39].

The prognostic value of PDGF reported in the literature is controversial. In a series of 98 patients with localised or metastatic PDAC, Rahbari et al. reported that elevated serum PDGF‐AA levels were associated with poorer survival rates, highlighting its potential role as a negative prognostic marker [33]. In another study, Kahlert et al. applied a compartment‐specific profiling approach to assess multiple markers in PDAC, distinguishing between tumour epithelium, tumour stroma and patient serum. The key findings revealed that high expression of tumour PDGF‐BB correlated significantly with prolonged survival and was confirmed as an independent prognostic factor in multivariate analysis [21]. The significant negative impact of PDGF‐A on survival in patients with PDAC was also reported in a study based on analysis of gene expression in tumour samples using laser microdissection and real‐time reverse transcription polymerase chain reaction techniques [40].

Regarding the prognostic impact of PDGF in CCA, a study exploiting both in vitro (cell‐based) and in vivo (animal‐based) experimental models and human samples showed that PDGF was a key molecule in boosting the invasive and metastatic capabilities of CCA cells by upregulating the expression of MMP‐2 and MMP‐9 [19]. In the same direction, in a series of 20 patients with CCA, Luo et al. reported that PDGF‐CC serum levels were significantly elevated compared with healthy controls and that tumour‐expressed PDGF‐CC was independently associated with poor patient survival [18]. In addition, it has been recently reported that the interactions between PDGF and cancer‐associated fibroblasts (CAFs) were a major event in CCA invasiveness. A study using both laboratory cell experiments and animal models demonstrated that PDGF‐D contributed to the progression of CCA by activating CAFs, which in turn stimulated lymphangiogenesis through the release of VEGF‐A and VEGF‐C. This process was closely linked to poor prognosis due to early tumour metastasis [17]. Focusing on CAF, Yan et al. highlighted that CAFs, through the PDGF‐BB/PDGFR‐β signalling pathway, played a significant role in promoting lymphatic metastasis in CCA [20].

One of the few studies on bile, conducted on both PDAC and CCA, showed that PDGF‐AB biliary levels were significantly higher in patients with cholangiocarcinoma than in those with PDAC or benign diseases and that higher PDGF‐AB levels were linked to a more active or rapidly growing tumour [23]. In our study, we also found that the biliary concentration of PDGF‐AA was significantly higher in the CCA/PDAC group compared with the benign group. The diagnostic value was moderate, with an AUROC of 0.64. For the best cut‐off, the sensitivity was good at 81%, but the specificity was poor at 44%, precluding its use for implementation in clinical practice. In addition, high levels of biliary PDGF‐AA were associated with a better prognosis, in line with the data already reported on PDGF‐BB in the studies cited above. The discrepancies between studies may be due to differences in the specific PDGF isoform measured, the type of cancer studied (we considered both CCA and PDAC), or the nature of the biological samples analysed (e.g., bile, serum and tissue).

Biliary IGF‐1 has been reported to be a useful marker for the diagnosis of malignant obstruction. Abdel‐Razik et al. [14] showed that biliary levels of IGF‐1 were significantly increased in a malignant group of patients with PDAC or CCA compared with patients with benign biliary lesions. In a study by Alvaro et al., the biliary IGF‐I concentration was 15‐ to 20‐fold higher in extrahepatic cholangiocarcinoma than in pancreatic cancer or benign biliary abnormalities [15]. In our study, we did not confirm these results as biliary IGF had neither a diagnostic nor a prognostic value, thus ruling it out as a useful marker. However, owing to the significant number of missing values for this marker, our negative results should be interpreted with caution. Serum bilirubin was found to be significantly associated with an increased risk of malignancy in univariate and multivariate analysis. However, the level of serum bilirubin is directly related to the severity of biliary obstruction, which depends on the selection of patients in benign and malignant groups, and not to the nature of obstruction. As the level of cholestasis was significantly different between these groups in our study, serum bilirubin was included in the multivariate analysis to neutralise its potential confounding effects.

Nevertheless, there are several limitations in this study. First, the size of our patient series was relatively small, so that subgroup analyses were constrained by the limited number of patients. Second, because this study was retrospective and conducted in patients with an established final diagnosis, the performance of the ELISA‐based biomarkers in truly indeterminate biliary strictures at the time of ERCP could not be assessed and warrants dedicated prospective evaluation. Third, the lack of concomitant biomarker measurement in serum did not allow us to analyse the potential concordance with bile concentrations. The strengths lie in the high precision of the data and the very long longitudinal follow‐up, which allowed us to obtain detailed information about the diagnosed pathology and the clinical history of the patients.

In conclusion, this study provides valuable insights into the diagnostic and prognostic potential of various biomarkers for malignant biliary tract stenoses. The analysis reveals that biliary VEGF is an independent diagnostic marker of malignant biliary obstruction due to PDAC or CCA in both univariate and multivariate analyses. In addition, MMP‐2 shows an independent association with CCA but not PDAC diagnosis. The findings of this study align with those of previous reports, highlighting the importance of VEGF as a diagnostic marker of malignant biliary obstruction. However, the results also indicate limitations in differentiating between PDAC and CCA using the analysed biomarkers alone, suggesting the need for further investigation into more specific markers or their combinations. While some biomarkers, such as MMP‐1, MMP‐2 and IGF, showed initial promise in univariate analysis, they did not maintain significance in multivariate models. This underscores the complexity of biomarker research and the importance of rigorous statistical analyses in identifying truly independent predictors.

These results contribute to the growing body of evidence on biliary and serum biomarkers of malignant biliary tract tumours. However, further research with larger sample sizes and prospective designs is required to validate these findings and explore their clinical applicability. Additionally, investigating combinations of biomarkers and integrating them with other clinical and imaging parameters may enhance their diagnostic and prognostic utility in clinical practice.

## Author Contributions

E.G., and G.T., had full access to all data in the study and took responsibility for data integrity and the accuracy of data analysis. G.T., R.G., and G.D.S.: study concept and design. G.T., V.U., R.G., and S.A.: acquisition of data. E.G., G.T., and S.C.: analysis and interpretation of data. E.G., G.T., R.G., and G.D.S.: drafting of the manuscript. G.T., E.G., G.D.S., R.R., C.B.‐R., and L.G.: critical revision of the manuscript. S.C., and E.G.: statistical analysis. G.T.: obtained funding. V.U., and R.G.: administrative, technical and material support. G.T.: study supervision.

## Funding

This work was supported by CHU Reims. This project received funding from the local research grant call issued by the University Hospital of Reims (CHU de Reims).

## Conflicts of Interest

The authors declare no conflicts of interest. For transparency, the following general disclosures are reported by E.G.: Honoraria, Gilead, AbbVie, AstraZeneca, Servier, Roche. Consulting/Advisory Roles: Sanofi, Sandoz, AbbVie, AstraZeneca. Travel/Accommodations/Expenses: Bayer, Gilead, Roche, AbbVie, Ipsen, Janssen, Galapagos, Pfizer, Orphalan.

## Supporting information


**Figure S1:** Comparison of MMPs (A,B,C,D), VEGF (E), PDGF‐AA (F) and IGF (G)biliary concentrations in benign and malignant groups. **Figure S2:** Principal component analysis (PCA). A. PCA with two principal components (PC1 and PC2) described 48% of the overall variance. In the resulting graph, patient data were divided into three groups: benign obstructions (black circles), cholangiocarcinoma (CCA) (red circles) and pancreatic ductal adenocarcinoma (PDAC) (green circles). B. Scree Plot d*escribing* the variance explained by each component of the full feature set. **Figure S3:** ROC curves of MMP‐7 values in differentiating patients with malignant versus benign obstruction. **Figure S4:** Correlation graph. The variables included in the graph were serum bilirubin concentration, MMP‐7, MMP‐1, VEGF, MMP‐2, PDGF‐AA, IGF, biliary bilirubin concentration, age and MMP‐9 levels. Each cell in the matrix represents the correlation between the two variables. The numbers indicate the strength and direction of the correlation, with values close to 1 indicating a strong positive correlation. Values close to −1 indicate a strong negative correlation between the two variables. Values close to zero indicate no correlation.
**Table S1:** Univariate and multivariate analyses: variable association with PDAC diagnosis in comparison with CCA.
**Table S2:** Sensitivity concordance and cumulative value associated with VEGF measurement and bile cytology analysis.

## Data Availability

The data that support the findings of this study are available from the corresponding author upon reasonable request.
