# Supplementary material for: Diagnostic and Prognostic Values of Bile Biomarkers for Malignant Biliary Stenosis
Source: Liver Int. 2026 Apr 21;46(5):e70637. doi: 10.1111/liv.70637 (PMC13100460; doi:10.1111/liv.70637)
Supplement: Supplementary file 1 — Figure S1: Comparison of MMPs (A,B,C,D), VEGF (E), PDGF‐AA (F) and IGF (G)biliary concentrations in benign and malignant groups. Figure S2: Principal component analysis (PCA). A. PCA with two principal components (PC1 and PC2) described 48% of the overall variance. In the resulting graph, patient data were divided into three groups: benign obstructions (black circles), cholangiocarcinoma (CCA) (red circles) and pancreatic ductal adenocarcinoma (PDAC) (green circles). B. Scree Plot describing the variance explained by each component of the full feature set. Figure S3: ROC curves of MMP‐7 values in differentiating patients with malignant versus benign obstruction. Figure S4: Correlation graph. The variables included in the graph were serum bilirubin concentration, MMP‐7, MMP‐1, VEGF, MMP‐2, PDGF‐AA, IGF, biliary bilirubin concentration, age and MMP‐9 levels. Each cell in the matrix represents the correlation between the two variables. The numbers indicate the strength and direction of the correlation, with values close to 1 indicating a strong positive correlation. Values close to −1 indicate a strong negative correlation between the two variables. Values close to zero indicate no correlation. Table S1: Univariate and multivariate analyses: variable association with PDAC diagnosis in comparison with CCA. Table S2: Sensitivity concordance and cumulative value associated with VEGF measurement and bile cytology analysis. [file LIV-46-0-s001.docx]

**Supplementary Materials:**

**Bile analysis methods details**

Assays for each biomarker were performed according to the manufacturer’s instructions. The measurements were performed with an adapted dilution realized in NaCl 0.9% (Miniversol, Dutscher, France) of the samples, except for MMP-2, which was measured without dilution. All assays were realized in 3 repeat wells of the same kit to determine intra-assay variability. The optical density of all samples was measured at 450 nm using a microplate reader.

**Measurement of bilirubin concentration**

Total bilirubin in the bile supernatant was measured using a diazo colorimetric method with a COBAS 8000 analyzer (Roche Diagnostics/Hitachi, France) according to the manufacturer’s instructions. For the measurement, the samples were 5-fold diluted with NaCl 0.9% (Miniversol, Dutscher, France). Before analysis, all samples were stored away from direct light to avoid possible degradation of bilirubin.

**Supplementary figure 1. Comparison of MMPs (A,B,C,D), VEGF (E), PDGF-AA (F) and IGF (G)biliary concentrations in benign and malignant groups.**

**B**

**A**

**C**

**D**

**F**

**E**

**G**

**Supplementary Figure 2. Principal component analysis (PCA)**

1. PCA with two principal components (PC1 and PC2) described 48% of the overall variance. In the resulting graph, patient data were divided into three groups: benign obstructions (black circles), cholangiocarcinoma (CCA) (red circles), and pancreatic ductal adenocarcinoma (PDAC) (green circles).
2. Scree Plot describing the variance explained by each component of the full feature set

# Supplementary figure 3. ROC curves of MMP-7 values in differentiating patients with malignant versus benign obstruction

**
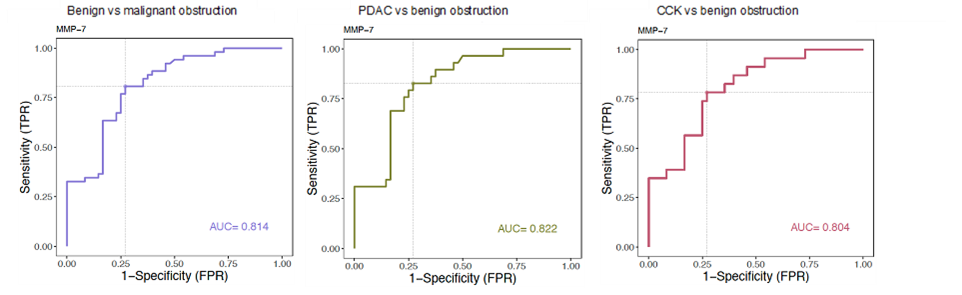
**

**Supplementary Figure 4. Correlation graph.** The variables included in the graph were serum bilirubin concentration, MMP-7, MMP-1, VEGF, MMP-2, PDGF-AA, IGF, biliary bilirubin concentration, age, and MMP-9 levels. Each cell in the matrix represents the correlation between the two variables. The numbers indicate the strength and direction of the correlation, with values close to 1 indicating a strong positive correlation. Values close to -1 indicate a strong negative correlation between the two variables. Values close to zero indicate no correlation.


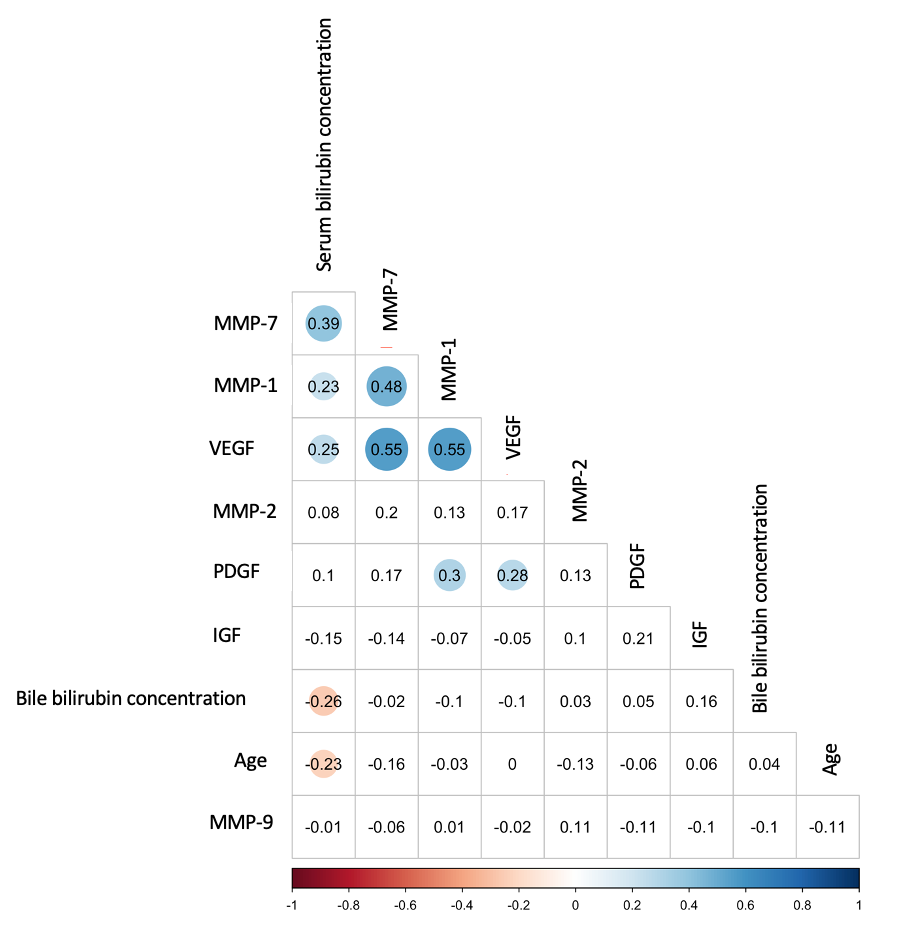


**Supplementary Table 1.** Univariate and multivariate analyses: variable association with PDAC diagnosis in comparison with CCA.

|  |  | UNIVARIATE | | | MULTIVARIATE | | |
| --- | --- | --- | --- | --- | --- | --- | --- |
| Variable | Available data | P-value | OR | CI | P-value | OR | CI |
| Male sex | 52 | 0.1392 | 2.5412 | 0.7383 - 8.7468 | - | - | - |
| Age | 52 | 0.7742 | 0.9918 | 0.9376 - 1.0492 | - | - | - |
| Serum bilirubin | 50 | 0.9325 | 1.0002 | 0.9960 - 1.0044 | - | - | - |
| Bile bilirubin | 52 | 0.2181 | 0.9996 | 0.9990 - 1.0002 | - | - | - |
| MMP-1 | 52 | 0.7054 | 1.0026 | 0.9894 - 1.0159 | - | - | - |
| MMP-7 | 52 | 0.7163 | 0.9994 | 0.9959 - 1.0028 | - | - | - |
| VEGF | 52 | 0.0742 | 1.5415 | 0.9585 - 2.4789 | - | - | - |
| MMP-2 | 51 | 0.4134 | 1.1054 | 0.8694 - 1.4053 | - | - | - |
| MMP-9 | 52 | 0.2538 | 1.0032 | 0.9977 - 1.0088 | - | - | - |
| PDGF-AA | 42 | 0.8544 | 1.0733 | 0.5039 – 2.2864 | - | - | - |
| IGF | 42 | 0.3015 | 0.5363 | 0.1645 - 1.7487 | - | - | - |

**Supplementary Table 2.** Sensitivity concordance and cumulative value associating VEGF measurement and bile cytology analysis.

|  |  |
| --- | --- |
| **Whole population ( PDAC +CCA), n=52** |  |
| True positive VEGF / False negative cytology | 22 |
| False negative VEGF / true positive cytology | 5 |
| True positive VEGF or cytology | 50 |
| Sensitivity VEGF or cytology | 96.2% |
|  |  |
| **PDAC patients (n=29)** |  |
| True positive VEGF / False negative cytology | 15 |
| False negative VEGF / true positive cytology | 2 |
| True positive VEGF or cytology | 29 |
| Sensitivity VEGF or cytology | 100% |
|  |  |
| **CCA patients (n=23)** |  |
| True positive VEGF / False negative cytology | 7 |
| False negative VEGF / true positive cytology | 3 |
| True positive VEGF or cytology | 21 |
| Sensitivity VEGF or cytology | 91.3% |
